# Erroneously Disgusted: fMRI Study Supports Disgust-Related Neural Reuse in Obsessive-Compulsive Disorder (OCD)

**DOI:** 10.3389/fnbeh.2019.00081

**Published:** 2019-04-24

**Authors:** Kathrin Viol, Benjamin Aas, Anna Kastinger, Martin Kronbichler, Helmut Johannes Schöller, Eva-Maria Reiter, Sarah Said-Yürekli, Lisa Kronbichler, Brigitte Kravanja-Spannberger, Barbara Stöger-Schmidinger, Wolfgang Aichhorn, Guenter Karl Schiepek

**Affiliations:** ^1^Institute for Synergetics and Psychotherapy Research, Paracelsus Medical University, Salzburg, Austria; ^2^Department of Psychosomatics and Inpatient Psychotherapy, Paracelsus Medical University, Salzburg, Austria; ^3^Department of Psychology, Ludwig Maximilian University of Munich, Munich, Germany; ^4^Department of Child and Adolescent Psychiatry, Psychosomatics and Psychotherapy, University Hospital, Ludwig Maximilian University, Munich, Germany; ^5^Department for Psychosomatics and Inpatient Psychotherapy, Paracelsus Medical University, Salzburg, Austria; ^6^Centre for Cognitive Neuroscience, University of Salzburg, Salzburg, Austria; ^7^Neuroscience Institute, Christian Doppler Clinic, University Hospital Salzburg, Salzburg, Austria; ^8^Department for Radiotherapy and Radio-Oncology, Christian Doppler Clinic, University Hospital Salzburg, Salzburg, Austria; ^9^Department of Neurology, Christian-Doppler Medical Center, Paracelsus Medical University, Salzburg, Austria

**Keywords:** OCD, fMRI, disgust, insular cortex, precuneus, neural reuse, picture rating

## Abstract

**Objective**: fMRI scans of patients with obsessive-compulsive disorder (OCD) consistently show a hyperactivity of the insular cortex, a region responsible for disgust-processing, when confronted with symptom-triggering stimuli. This asks for an investigation of the role of disgust and the insula in OCD patients.

**Methods**: Seventeen inpatients with OCD and 17 healthy controls (HC) underwent fMRI scanning. Whole-brain contrasts were calculated for “Disgust vs. Neutral” for both groups, plus an analysis of variance (ANOVA) to assess the interaction between group and condition. Additionally, the emotional dimensions of valence and arousal, along with the ability to cope, were assessed by picture ratings.

**Results**: The picture ratings confirmed the patients’ heightened sensitivity to disgust with higher values for arousal and inability to cope, but not for valence. fMRI scans revealed no hyperactivity of the insula in patients compared to controls for the condition “Disgust vs. Neutral,” indicating no basic hypersensitivity to disgusting stimuli. Increased activity in the precuneus in controls for this condition might correspond to the downregulation of arousal.

**Conclusions**: The absent differences in neural activity of the insula in patients compared to controls for the disgust-condition, but heightened activity for symptom-provoking conditions, suggests that the illness is due to an erroneous recruitment of the insula cortex for OCD-stimuli. The finding is interpreted within the framework of the neural reuse hypothesis.

## Introduction

With a prevalence of 2%–3%, obsessive-compulsive disorder (OCD) is one of the most common psychiatric diseases and has a serious impact on the quality of life (Crino et al., 2005). Individuals with OCD experience a persistent intrusion of unwanted thoughts or images (obsessions) and/or the urge for repetitive, ritualistic behaviors or mental acts (compulsions) that need to be neutralized in order to reduce anxiety or distress (American Psychiatric Association, 2013). Although the etiology of OCD has not been fully understood yet, a line of research suggests that disgust proneness, i.e., a disposition or personality trait towards enhanced reactivity to disgust, may play an important role (Olatunji et al., 2011). The theory has been backed by empirical studies revealing elevated self-reported ratings for disgust in clinical and non-clinical samples (e.g., Olatunji et al., 2011; Berle and Phillips, 2006; Whitton et al., 2015). The aim of this study was to investigate if a heightened sensitivity to disgusting pictures can also be found in our sample and if this hypersensitivity corresponds to differences in disgust processing in the brain in OCD patients. We were especially interested in the role of the insular cortex since this region has been identified as relevant for disgust processing (next to a variety of other functions) in patients as well as in healthy participants. Group differences were found in both functional imaging and lesion studies (e.g., Wright et al., 2004; Knowles et al., 2018), especially when highly disgusting stimuli were used (Oaten et al., 2018).

Next to its response to disgust, the insular cortex is commonly activated in OCD patients when confronted with OCD-related stimuli. These OCD-related pictures are designed to provoke the symptoms of patients and show situations that OCD patients experience as triggering but are commonly perceived as neutral by healthy participants. For example, a clean toilet is often reported to provoke the patients’ urge to continue cleaning (in the disgusting pictures condition, in contrast, a dirty toilet would be shown that provokes emotions of disgust also in healthy participants). Numerous studies have reported the insula’s activation in such OCD-related conditions in patients but not in controls (e.g., Schienle et al., 2005; Schiepek et al., 2013; Thiel et al., 2014), especially also for the sample of this study (Viol et al., 2019). This suggests that the hypersensitivity to disgust in the pictures ratings of OCD patients corresponds to a hyperactivity of the insular cortex. However, the difference in the activity of the insula between patients and controls in a pure disgust-based paradigm revealed heterogeneous results. While Schienle et al. (2005) and Berlin et al. (2015) found greater activation of the insula in response to disgust-inducing images in OCD patients compared to controls, no such group difference was found in Thiel et al. (2014) and Weygandt et al. (2012). Moreover, no correlation could be found between OCD symptom severity and insular activity (e.g., Berlin et al., 2017). Overall, it is still unclear if OCD is related to abnormal disgust processing, and if a general hyperactivity of the insular cortex might cause the patients’ hypersensitivity to disgust.

A note should be made on the heterogeneity of situations that provoke symptoms in OCD patients. Of course, a possible overlap between disgust-reaction and OCD-symptoms seems obvious for patients from the washing subtype, who fear contamination by disgust-related stimuli in the environment. However, it has been suggested that disgust may also play a fundamental role for the other OCD subtypes, i.e., symmetry/ordering, hoarding, and checking (e.g., Thorpe et al., 2003; Taylor and Liberzon, 2007). This observation makes sense in the context that feelings of disgust may not only arise from sensory experiences (e.g., taste, smell), but also from more abstract concerns, e.g., moral judgments (Rozin et al., 1999). Increased scores of disgust ratings also correlated with self-reported checking, ordering, neutralizing, and obsessing symptoms and even for the hoarding subtype (Olatunji et al., 2011). The findings remained significant after controlling for heightened anxiety, depression, and general fearfulness (Olatunji et al., 2005, 2011) and speak in favor of the hypothesis that the primarily “protective function of disgust has been extended from physical to psychological contamination” (Olatunji et al., 2011, p. 933). In conclusion, a possible hypersensitivity to disgust can not only be postulated for patients from the washing/contamination-subtype, but might be a general aspect of the illness.

As hypotheses, heightened scores were expected for patients in comparison to controls for the picture ratings for disgusting pictures in all dimensions (valence, coping and arousal), representing a more negative perception and emotional response. Based on the suggested biological hypersensitivity to disgust of OCD patients, we expected heightened neural activity in the insular cortex compared to controls for the contrast “disgusting vs. neutral pictures.”

## Materials and Methods

### Study Procedures

Within the 1st week of hospitalization, patients underwent an fMRI scan, followed by a picture rating including the emotional dimensions valence, arousal, and coping of the pictures seen in the scanner. The controls were shown the pictures of their respective patients and also rated the pictures afterward. Clinical symptoms were assessed with the Yale-Brown Obsessive Compulsive Scale (Y-BOCS, Goodman et al., 1989; German version: Hand and Buettner-Westphal, 1991), the Symptom Checklist-90-R (SCL-90-R, Derogatis et al., 1977; German version: Glöckner-Rist and Stieglitz, 2012), the Depression-Anxiety-Stress Scales (Lovibond and Lovibond, 1995; German version: Nilges and Essau, 2015), and the Beck Depression Inventory II (BDI-II, Beck et al., 1996; German version: Hautzinger et al., 2009).

### Participants

The sample consisted of 17 inpatients [six men and 11 women, mean age 43.5 years (SD = 10.7)], receiving an integrative treatment approach including cognitive-behavioral components at the Christian-Doppler University Hospital, Salzburg, Austria, as well as 17 healthy controls (HC) matched by age and gender. Patients were eligible to participate in the study if OCD was the main illness by clinical judgment based on ICD-10 and DSM-IV criteria and on the Structured Clinical Interview for DSM-IV Axis I disorders (SCID-I, First et al., 2002). Exclusion criteria consisted of neurological impairment and/or neurological diseases, acute psychosis, substance abuse, and/or suicidality. The mean Y-BOCS score of 26.7 (SD = 8.8) ranks the sample on the medium to the upper end of symptom severity. Comorbidities, as commonly found in OCD patients, included depression (eight patients), social phobia (two patients, in addition to depression) and one each from the schizophrenic spectrum, alcohol and substance abuse (currently abstinent), and posttraumatic stress disorder (PTSD). The mean BDI-II score of patients was 29 (SD = 9.4) and 1.2 (SD = 1.5, *p* < 0.001) for controls. All but one patient took some kind of antidepressant (mostly SSRI), seven of them in addition neuroleptics, three anticonvulsants, two benzodiazepine and one lithium. One patient also had to be medicated for high blood pressure, thyroid dysfunction and incontinence. The study was approved by the Ethics Commission Salzburg (Ethikkommission Land Salzburg, No. 415-E/1203/5-2012). Detailed information on the study was provided and written informed consent was obtained from all participants according to the Declaration of Helsinki.

### Stimuli

**Figure 1:**
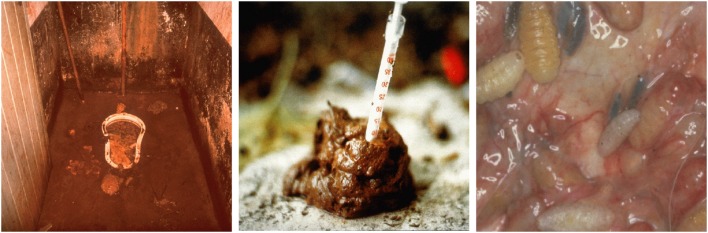
Examples of disgusting pictures used as stimuli in the fMRI scans from the International Affective Pictures Set (IAPS, Lang et al., 2008).

For symptom provocation during the fMRI scan, patients as well as controls were shown colored pictures from four categories, namely individual OCD-related photos, standardized OCD-related photos from the Maudsley Obsessive-Compulsive Stimulus Set (MOCCS, Mataix-Cols et al., 2009), and disgusting and neutral pictures from the International Affective Pictures Set (IAPS, Lang et al., 2008). For details on the acquisition and selection process of the individual pictures see Viol et al. (2019). Examples of the disgusting pictures are depicted in Figure 1. Participants were instructed to passively view the pictures.

In the scanner, the pictures were displayed on an MRI compatible LCD screen by Conrac (distributed by Siemens) placed at the back of the MRI bore with a size of 18 inch and a resolution of 1,280 × 1,024 pixel. Participants were able to see the pictures on a mirror mounted directly above the participants’ heads on the head coil in 145 mm distance. The original picture size was 750 × 750 pixel.

### Picture Rating

Immediately after the fMRI scan, participants rated the pictures they have just seen in the scanner. Ratings were conducted computer-based with the E-Prime 2.0 presentation software^1^. First, the concept of valence and arousal as the two dimensions of emotions (Bradley et al., 1992) and coping (i.e., how well the participants felt that they could handle the situation) was explained to the patients, followed by a short introduction and training on the software. Two-sided *t*-tests were calculated for differences in groups and corrected for false discovery rates (FDR).

### fMRI Data Acquisition

Images were acquired with a 3T Siemens TIM TRIO whole-body scanner (Siemens Symphony, Erlangen, Germany) with a 32-channel head coil. First, a high-resolution scan was acquired for anatomical referencing using a T1-weighted MPRAGE sequence (FoV: 256 mm, slice thickness: 1.0 mm, TR: 2,300 ms, flip angle: 9°, resolution: 1 × 1 × 1 mm). Functional images were obtained in two sessions with a short pause in between. Concerning the functional imaging, a total of 552 volumes were acquired using a T2*-weighted gradient echo EPI with 36 slices (slice thickness: 3 mm, descending slice order, TR: 2,250 ms, TE: 30 ms, flip angle: 70°, FoV: 192 mm). The first six volumes of each functional session were discarded due to saturation effects (Sarty, 2007), leaving a total of 540 volumes. Stimuli were presented with the E-Prime 2.0 presentation software^1^ as an event-related design in a pseudo-randomized order in two sessions (20 pictures of each category in each session). The pictures were shown for 4 s each, separated by a fixation cross; the inter-stimulus interval was 2 s. The resulting DICOM files were converted to 4D-NIfTI-files with the tools MRIConvert^2^ and dcm2nii^3^.

### Preprocessing

Preprocessing and statistical analysis were performed using the Statistical Parametric Mapping software package SPM12 (Wellcome Department of Cognitive Neurology, London, UK) implemented in Matlab (Mathworks, Inc., Natick, MA, USA, release 13a). Functional images were realigned to the first image, de-spiked with the AFNI 3d-despike function^4^, unwarped, corrected for geometric distortions using the field map of each participant, and slice time corrected.

The high resolution structural T1-weighted image of each participant was processed and normalized with the CAT12 toolbox^5^ using default settings. Each structural image was segmented into gray matter, white matter and CSF, and denoised, then warped into MNI space by registration to the DARTEL template provided by the CAT12 toolbox *via* the high-dimensional DARTEL registration algorithm (Ashburner, 2007). Based on these steps, a skull-stripped version of each image in native space was created. To normalize functional images into MNI space, the functional images were coregistered to the skull stripped structural image and the parameters from the DARTEL registration were used to warp the functional images, which were resampled to 3 mm × 3 mm × 3 mm voxels and smoothed with a 6 mm FWHM Gaussian kernel. The quality of the preprocessing was checked using the tools BXH^6^ and tsdiffana^7^.

### Statistical Analysis

Since SPM uses a mass-univariate approach, the effects of the conditions were modeled for each voxel with a general linear model (Kiebel and Holmes, 2008). The movement parameters gained from the realignment procedure during preprocessing were used as regressors. A 2 × 2 analysis of variance (ANOVA) was calculated using the Multivariate and Repeated Measures (MRM)^8^ toolbox for MATLAB provided by the University of Manchester. A within-subject factor (disgust and neutral pictures) was modeled along with a between-subject factor (patients and controls). Unless otherwise stated, *p*-values have been corrected for multiple comparisons (FWE_Peak_-correction).

A ROI analysis of the left and right insula was performed to further investigate the differences in activation between groups. In addition, the betas, i.e., the estimated β-parameters from the general linear model, were extracted for each participant. The values were extracted for the contrast “Disgust vs. Neutral” at the left insular cortex, using the voxel with maximal *T*-value (Table 2) at group level.

**Table 1:** Arithmetic mean values of the picture rating for the dimensions valence, arousal and ability to cope.

Stimulus	Patients	Controls	*p*
Disgust			
Valence	3.08 (1.69)	3.51 (1.18)	0.48
Arousal	5.92 (2.09)	3.71 (2.47)	0.03*
Coping	4.63 (2.34)	6.85 (2.44)	0.03*

*p < 0.05. In brackets: SD. *p*: *p*-values of two-sided *t*-test with H_0_: mean (patients) = mean (controls), FDR-corrected.

**Table 2:** ROI analysis of the insula for the contrast “Disgust > Neutral” for patients and controls.

Group	L/R	*x*	*y*	*z*	*T*
Patients	L	−39	−1	−2	4.50*
Controls	L	−36	5	−17	5.93**
	R	27	11	−17	7.74***

*p < 0.0001 uncorrected with extent threshold = 10 voxels, **p < 0.05 FWE_Cluster_, ***p < 0.05 FWE_Peak_.

Note that the current study is part of a larger project including 4–5 scans during psychotherapy. The following sections report information and results relevant for the above-mentioned hypotheses only, i.e., data from the first fMRI-scans at the very beginning of the therapy, since the group differences should be greatest at this point.

## Results

### Picture Ratings

**Figure 2:**
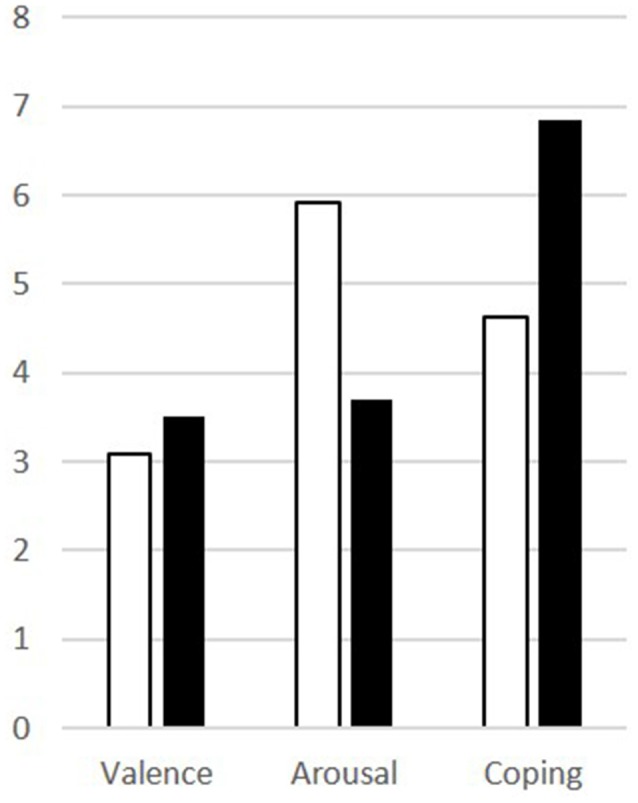
Results of the picture ratings for disgusting pictures for patients (white) and controls (black) for valence, arousal, and coping.

Patients scored significantly higher in arousal and assessed their ability to cope lower than controls for the disgusting pictures, but there was no difference in the rating of the valence (Table 1). The results are visualized in Figure 2.

Further investigation of the relationship between arousal and coping revealed a correlation of *r* = −0.99 for both patients and controls; an ANOVA confirmed no effect of group (*p* = 0). Coping can, therefore, be fully explained by arousal (*R*^2^ = 1).

### Whole-Brain Analysis

**Figure 3:**
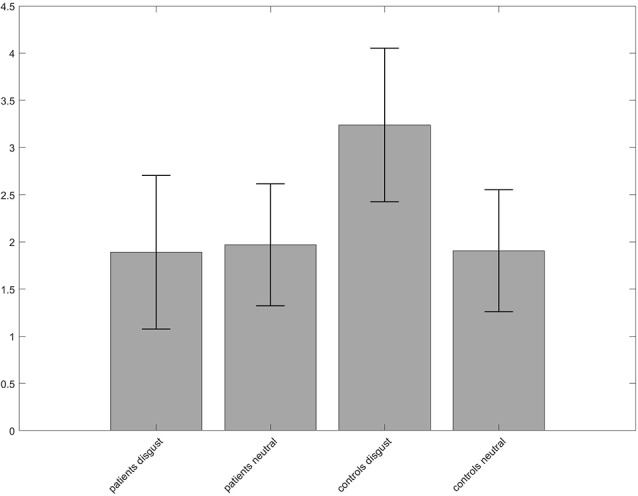
Group × condition interaction of the analysis of variance (ANOVA) revealed different activation in a cluster in the left precuneus. The contrast estimates of the peak voxel at MNI coordinates *x* = −24, *y* = −67, *z* = 40 show that neural activity of the controls was higher for the disgust condition compared to patients and to the neutral condition.

A 2 × 2 ANOVA on whole-brain level with the between-subject factor “group” (patients, controls) and the within-subject factor “condition” (disgust, neutral pictures) was performed to investigate the difference between groups and conditions. No interaction effect was found for *p* < 0.05 FWE-corrected on peak-level. When lowering the level of significance to *p* < 0.001 and using *p* < 0.05 FWE-corrected on cluster level, a cluster in the left precuneus/superior parietal lobe with a peak at [−24, −67, 40] became significant. The contrast estimates revealed a heightened activation in the disgust condition for the controls (Figure 3).

### ROI-Analysis of the Insula Cortex

To specifically investigate the role of the insula in disgust processing, a ROI analysis of the insula cortex was performed for patients and controls for the contrast “Disgust > Neutral” (Table 2). Significant activation of the left and right insula was found for the controls for *p* < 0.05 FWE (cluster level, based on *p*_peak_ < 0.001). For patients, the activity was found in the left insula only and did not survive FWE correction on peak or cluster level. No significant activation was found for the opposite contrast (“Neutral > Disgust”) for neither controls nor patients.

**Figure 4:**
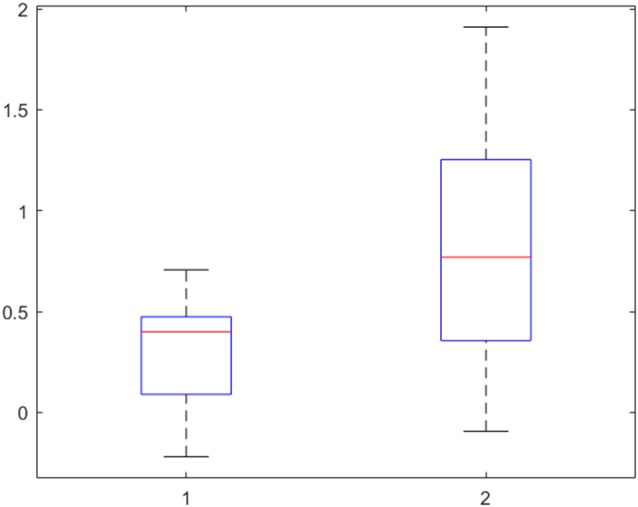
Beta-values of the left insular cortex in patients (left) and controls (right) for the contrast “Disgust vs. Neutral” reveal a lower neural activity, indicating no hyperactivity to disgust in obsessive-compulsive disorder (OCD) patients. 50% of the values are within the boxes, with the median marked as a horizontal line. The upper and lower 25% is indicated by the whiskers.

Finally, the activity in the left insular cortex of patients and controls was further explored with the beta values, interpretable as the height of neural activity, of each participant. The result is presented in Figure 4. A two-sided *t*-test confirmed the assumption from Table 2 that the activity in the left insular cortex of patients is indeed *lower* than that of controls (*p* = 0.002).

## Discussion

### OCD Affects Arousal and Ability to Cope Rather Than Emotional Valence of Disgust

**Figure 5:**
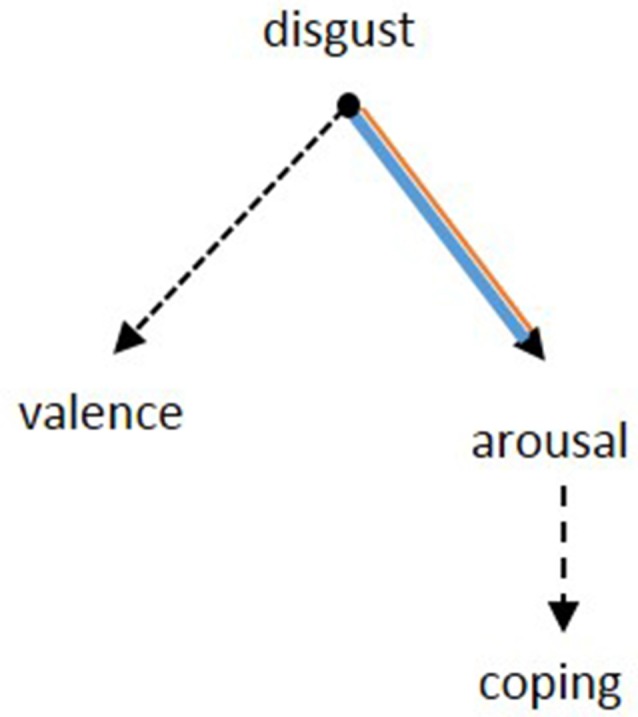
Based on the results from the picture rating, the pathology of OCD seems to be specified by the inability to suppress arousal after a confrontation with disgusting stimuli. Concerning the rating of valence, there was no difference between patients and controls. The patients’ lower ability to cope might be a consequence of the heightened arousal. Dotted lines: negative relation, continuous lines: positive relation. Black: no group difference, blue: patients, orange: controls. The width of the lines represents the strength of the correlations.

The heightened sensitivity of OCD patients to disgusting pictures has been shown frequently in picture ratings. Our results of the picture ratings confirm this finding and allow to further differentiate the emotional dimension of this hypersensitivity. Notably, the valence of the disgusting pictures, i.e., how pleasant the pictures were rated, did not differ between groups. While the *evaluation* of the pictures was comparable between patients and controls, the *reaction* of patients was indeed significantly different: patients were more aroused and felt less able to cope with the depicted situations. In other words, OCD sufferers perceive disgust-related situations no different to healthy people, but become more agitated and feel less able to deal with the situation due to the heightened arousal. It seems like the hypersensitivity might actually be a problem of regulating arousal (Figure 5).

### Group Differences in Neural Activity for Disgust

The differences in neural activation between patients and controls for disgusting stimuli compared to neutral pictures was assessed in two ways, first by an ANOVA on whole-brain level, i.e., assessing all voxels in the brain. Second, a ROI analysis was conducted for the insula cortex, which is known to be involved in disgust processing.

The whole-brain ANOVA did not reveal any different neural activity when the correction for multiple comparisons (FWE) was applied to each voxel. If the threshold was lowered to *p* < 0.001 per voxel and the FWE-correction was performed for clusters, heightened activation was found for the left precuneus in controls for the disgust condition. The precuneus is known for several functions concerning general emotional processing, but not specific for disgust. It rather seems that the region is involved in emotion regulation, as it was linked to the intensity of emotions (Sato et al., 2015). Bestelmeyer et al. (2017) found activity in the precuneus for both valence and arousal, suggesting a modulating role. Linking these results with the heightened arousal reported by patients in the picture rating, one might suggest that the precuneus’ activity is a neural correlate of the *suppression* of arousal in controls. Similar results have been reported by Ashworth et al. (2011), where patients with bulimia nervosa showed reduced activity in the precuneus when confronted with facial expressions of disgust and anger compared to controls. In conclusion, the role of the precuneus in emotion processing might be relevant for several psychiatric disorders but does not seem to be specific for disgust or OCD.

The ROI analysis of the insula for the difference between disgusting and neutral pictures confirmed the region’s involvement in disgust processing. However, the group difference was not significant. An examination of the beta-values (interpretable as the neural activity at a specific voxel) shows *increased* activity in controls compared to patients. This is in opposition to our hypothesis of a hyperactivity in the insula in patients as an underlying mechanism for OCD. In other words, no basic neural hypersensitivity to disgust can be found in patients.

### The Neural Reuse Hypothesis

What remains is the question of why the insular cortex is consistently reported as hyperactive in symptom-provoking conditions in OCD patients compared to controls, but not in the disgust-condition. As reported in detail in Viol et al. (2019), a significant group difference (*p* < 0.05, FWE_Peak_-corrected) was also found in this sample in the insula for the individual symptom-provoking OCD-stimuli vs. neutral pictures. Moreover, in the disgust condition, no abnormal activity was found in any of the regions commonly hyperactive in OCD, neither in the ACC, which is supposed to play the role of a conflict monitoring unit, nor in the frontal cortex, where executive functioning, impulse control and emotion regulation is assumed to take place. It can, therefore, be excluded that a general hypersensitivity of the insular cortex is responsible for heightened disgust-proneness in OCD patients. Still, its activation in symptom-provoking situations like standing in front of a clean toilet causes the neurons in the insula to fire more than in controls. It seems like a normally non-disgusting stimulus activates the insula in OCD patients, but not in controls. The patients “abnormal” insular response to symptom-provoking, but not to disgusting pictures, begs an explanation, which might be provided by the neural reuse hypothesis. The theory of neural reuse was originally developed in the context of brain development. The concept is based on the observation that a fundamental organizational principle of the brain seems to consist of acquiring already existing neural circuits to accomplish a new task. It is used as a possible explanation for how the brain is able to deal with tasks that it was originally, i.e., from an evolutionary point of view, not developed for. Such tasks include the acquisition of language, i.e., pathways supporting primate tool use have been extended to human language (Anderson, 2010, 2016; Glenberg and Gallese, 2012). Further studies also linked mathematical operations (Dehaene and Cohen, 2007) and emotional reactions to abstract stimuli like disgusting words (Ziegler et al., 2018) to this mechanism. By this, the theory is closely related to the idea of embodied cognition and mirror neurons (Gallese, 2008); a theoretical framework based on dynamic systems theory, and the reuse of common attractors was proposed by Candadai and Izquierdo (2018). Note that the idea differs from the concept of neural plasticity insofar as is does not (only) focus on lesions or loss of brain function, but suggests that new uses can be acquired to be used next to its primary purpose if a suitable function or structure for a particular new task is already existent in the brain. The recycling of a given system is therefore supposed to happen with only minor changes to the original structure, e.g., by establishing a functional connection to different regions, and without altering the original functionality (Anderson, 2010).

So far, neural reuse is used to explain a normal development, i.e., how the brain’s structure and function are able to adapt to new tasks in the environment by searching (on a macroscopic level) its existing neural circuits that might fit to solve the problem. This search mechanism is yet unknown, but most likely based on a probabilistic evaluation of patterns (Lu, 2016). Naturally, this process is not error-save and might go wrong. With reference to OCD, a disease often triggered by stressful or traumatic life events (Real et al., 2011), this could mean that in search of a way to deal with this event, the brain erroneously comes to the conclusion that the disgust-mechanism is helpful, e.g., by providing a possibility to regain the feeling of control. In consequence, non-disgusting stimuli like the hands that have just been cleaned are still assessed as contaminated, i.e., these stimuli are now associated with the neural circuits for disgust processing. The aberrant focus on the disgust processing units of the brain might be governed or mediated by genetic and/or neurochemical abnormalities frequently found in OCD sufferers and their siblings (Hettema et al., 2001). Since neural reuse is thought to influence the neural plasticity on a local level (Hebbian learning, Anderson, 2016), OCD can manifest and become chronic.

The principle has recently been shown to be valid for the emotion of disgust insofar that the insular cortex is activated by reading disgust words—which themselves are of course not disgusting (Ziegler et al., 2018). Further evidence is provided by our finding of heightened values in the picture ratings and the involvement of the insula as a neural correlate of OCD, i.e., disgust does indeed play an important role in OCD. However, the missing group difference in the neural activation in the disgust-condition shows that the basic processing of disgust-related stimuli is *not* altered in OCD. One might, therefore, speculate that the insula—while functioning normally for disgust—is falsely recruited during the development of OCD and is now active in non-disgusting, but OCD-specific situations, too. This is supported by findings of Belin-Rauscent et al. (2016), who showed that individual vulnerability to compulsive behavior in rats is based on abnormalities in the anterior insula.

To conclude, the neural reuse hypothesis suggests that a primary neural activity (disgust processing) is reused by patients in situations that are *not* disgusting. On the one hand, this can explain why OCD-patients from the washing subtype experience their just extensively cleaned hands/items as still disgusting, and on the other hand, why disgust is also relevant for OCD patients from the other subtypes. For the latter, the fear of losing control and harming oneself and/or others lays behind the compulsions to check and control. Accompanying obsessive thoughts focus on the possibility of being aggressive, engaging in unwanted sexual behaviors, violating religious rules, or otherwise engaging in immoral behavior. Such morally unacceptable behavior elicits what is described as “moral disgust,” i.e., its original functionality has been expanded on the psychological as well as on the neural level. Last but not least, the theory might even explain the result of lower insular activity in patients than controls: by being recruited for OCD-specific stimuli, which are present in patients during considerable hours of the day, the region is highly occupied and might not have enough resources left for other tasks like normal disgust-processing.

### Limitations

Some limitations have to be taken into consideration with regard to this study. First, patients were not medication-naïve. Even though psychotropic drugs are specifically designed to alter neural activity, it can be assumed that—if the patient still meets the criteria for OCD—the drug was not able to change the disease-specific activation to a non-clinical level. Second, comorbidities, especially with major depressive disorder, are common in OCD (Schiepek et al., 2011). In consequence, it cannot be excluded that some of the pictures also provoked altered neural activation due to a comorbidity in depression.

Concerning the interpretation of the results, one has to acknowledge the fact that the insula seems to be involved in a variety of other cognitive and emotional processes. With our research design, it cannot be fully excluded that its activity is due to e.g., the processing of fear since there was no such control condition available. Still, prior research and the missing correlation with the factor “fear” of the DASS (not reported) suggests that the insula is actually responding to disgust in this paradigm.

### Future Research

The results of this study can be fully integrated in the neural reuse hypothesis, but are not able to fully answer all questions concerned with this hypothesis. These should be focused in future research and investigate e.g., the identification of a general “disgust processing system” of the brain. While we were able to show that OCD-related stimuli recruit neural circuits of the insular cortex, one could speculate that other brain regions are also involved in the processing of disgust. To analyze these possible additional regions would be important in order to determine if the disgust-related activation in OCD is due to an abnormal connectivity with the insula only, or if other connections are altered, too. In this context, it is also not clear which regions are erroneously connected to the insula, i.e., how it is recruited. In addition, to further understand the etiology of OCD, it would be important to develop a theory on *why* the disgust system is reused, e.g., what problems or tasks existed in the environment of patients that required the brain to recruit the disgust circuits, and/or if there is a genetic disposition. With regard to psychotherapy, ideas on how can this concept can be used in the development of therapies for OCD should be investigated. Last but not least, considering the concept as a possible explanation for other psychiatric disorders—as discussed for social anxiety disorder by Varlet et al. (2014)—might improve understanding and treatment.

## Conclusion

The study confirms prior findings on heightened reactivity to disgust in OCD patients, but only on the psychological level. In the fMRI scans, no difference was found in activation of the insula between patients and controls, suggesting a normal functioning of the basic disgust processing. However, the insular cortex, known to be relevant for disgust processing, is commonly activated during symptom-provoking conditions, suggesting an erroneous recruitment of neural disgust processing circuits. The finding is interpreted as a neural reuse “gone wrong.” The theory is especially appealing because it is based on an existing neural mechanism and goes in line with the economical/parsimonious aspect of evolution. Moreover, it provides a theoretical framework to integrate prior findings on the heightened sensitivity to disgust also for patients from subtypes other than washing.

## Ethics Statement

This study was carried out in accordance with the Ethics Commission Salzburg (Ethikkommission Land Salzburg, No. 415-E/1203/5-2012) with written informed consent from all subjects. All subjects gave written informed consent in accordance with the Declaration of Helsinki. The protocol was approved by the Ethics Commission Salzburg.

## Author Contributions

KV analyzed the data and wrote the manuscript. BA and AK took the individual pictures with the patients at their homes, conducted the psychological tests and interviews and realized the picture ratings. MK set up the fMRI procedure and helped to analyze and interpret the data and advised on the statistical analysis. HS performed the statistical analysis of the picture ratings. E-MR realized the fMRI scans. SS-Y and LK prepared the scripts for fMRI analyses. BK-S and BS-S recruited the participants and gave information about the study. WA supervised the study. GS designed and supervised the study. All authors interpreted the results, contributed to manuscript revision, read, and approved the submitted version.

## Conflict of Interest Statement

The authors declare that the research was conducted in the absence of any commercial or financial relationships that could be construed as a potential conflict of interest.

## Footnotes

^1^http://www.pstnet.com

^2^https://lcni.uoregon.edu/downloads/mriconvert/mriconvert-and-mcverter

^3^http://www.mccauslandcenter.sc.edu/crnl/tools

^4^https://afni.nimh.nih.gov

^5^http://dbm.neuro.uni-jena.de/cat

^6^https://wiki.biac.duke.edu/biac:xmlheader

^7^http://imaging.mrc-cbu.cam.ac.uk/imaging/DataDiagnostics

^8^https://www.click2go.umip.com/i/s_w/mrm.html
